# A new adaptive testing algorithm for shortening health literacy assessments

**DOI:** 10.1186/1472-6947-11-52

**Published:** 2011-08-06

**Authors:** Sasikiran Kandula, Jessica S Ancker, David R Kaufman, Leanne M Currie, Qing Zeng-Treitler

**Affiliations:** 1 Department of Biomedical Informatics, University of Utah, Salt Lake City, UT. USA; 2 Departments of Pediatrics and Public Health, Weill Cornell Medical College, New York, NY. USA; 3 Department of Biomedical Informatics, Columbia University, New York, NY. USA; 4 School of Nursing, University of British Columbia, Vancouver, BC. Canada; 5 Veteran Affairs Salt Lake City Health Care System, Salt Lake City, UT. USA

## Abstract

**Background:**

Low health literacy has a detrimental effect on health outcomes, as well as ability to use online health resources. Good health literacy assessment tools must be brief to be adopted in practice; test development from the perspective of item-response theory requires pretesting on large participant populations. Our objective was to develop a novel classification method for developing brief assessment instruments that does not require pretesting on large numbers of research participants, and that would be suitable for computerized adaptive testing.

**Methods:**

We present a new algorithm that uses principles of measurement decision theory (MDT) and Shannon's information theory. As a demonstration, we applied it to a secondary analysis of data sets from two assessment tests: a study that measured patients' familiarity with health terms (52 participants, 60 items) and a study that assessed health numeracy (165 participants, 8 items).

**Results:**

In the familiarity data set, the method correctly classified 88.5% of the subjects, and the average length of test was reduced by about 50%. In the numeracy data set, for a two-class classification scheme, 96.9% of the subjects were correctly classified with a more modest reduction in test length of 35.7%; a three-class scheme correctly classified 93.8% with a 17.7% reduction in test length.

**Conclusions:**

MDT-based approaches are a promising alternative to approaches based on item-response theory, and are well-suited for computerized adaptive testing in the health domain.

## Background

More than half of the US adult population has limited health literacy skills [1]. Low health literacy can limit a person's ability to communicate effectively with healthcare providers, comprehend physician's instructions and make informed health decisions. In addition, a strong correlation between low health literacy and poor health outcomes have been documented in a range of medical problems [1].

The goal of the consumer health informatics initiatives is to improve patients' comprehension of health information. This requires tools that identify deficiencies in disadvantaged patient populations. Several health literacy measures are available, including the Rapid Estimate of Adult Literacy of Medicine (REALM) [2], the Test of Functional Health Literacy in Adults (TOFHLA) [3], and the Newest Vital Sign (NVS) [4]. In addition, several other assessments focus on numerical skills or numerical self-efficacy [5-7]. However, no single assessment tool covers text reading skills, numerical skills, ability to use forms, tables, and graphs, oral communication skills, conceptual understanding of physiological health issues, and ability to understand and navigate health systems such as hospitals and insurance companies, which are just some of the domains considered to be relevant to health literacy [8-10].

Even this partial list suggests that a truly comprehensive health literacy assessment would be prohibitively burdensome for both the patient and the clinician. In order for testing to be useful in clinical practice or for patient-oriented informatics initiatives, it would have to be made as simple and short as possible. Abbreviated versions of REALM[11-13] and TOFHLA[14] have been proposed, although the applicability of methods used to shorten these tests on new tests remains to be investigated.

Our current work is part of a research program with an overall objective of developing a flexible health literacy instrument for older adults. A short and powerful assessment would be particularly useful for older adults, who: (a) are more likely to have low health literacy [10,15], (b) use health care services more often than younger people [16], and (c) are more likely to experience fatigue from taking an exhaustive literacy exam.

The task of health literacy assessment is similar to mastery tests used in educational and professional domains, in which a subject needs to be classified as master or non-master. Huynh[17] and van der Linden[18,19] have studied the problem of administering fixed-length mastery tests while minimizing the cost of misclassification of the subjects. However, in cases where the subjects exhibit clear mastery (or non-mastery) early termination of the tests would be beneficial. This would require variable-length tests in which the decision to continue or terminate testing can be made after each item. In one of the earliest attempt at a variable-length testing, Ferguson[20] proposed an approach based on Wald's sequential probability ratio[21]. However, in this approach all items were considered to be of equal difficulty. Kingsbury and Weiss[22], Reckase[23] and Lewis and Sheenan[24] have proposed methods that do not rely on this assumption and estimate each item's difficulty using item response theory (IRT).

Under IRT, the probability of a correct response to item *i*, by an examinee of estimated ability θ is calculated as:

where *α_i _*∈ [0, ∞), *b_i _*∈ (-∞,∞) and *c_i _*∈ [0,1] are item-specific parameters that represent the difficulty, discriminating power and guessing probability of item *i *respectively [25,26]. IRT requires the items to be pre-tested on a large population in order to estimate the probability that an item is answered correctly by examinees of different competency levels. Although IRT-based approaches provide excellent results when examinees need to be scored with precision on a continuous scale, this calibration can be complex and resource-intensive. Multi-stage testing approaches can relax the calibration size requirements of IRT but would still require calibration samples in the order of a few hundred subjects[27].

The methods proposed by Kingsbury and Weiss[22] and Reckase[23] have used decision theory principles to evaluate the stopping function i.e. the cost of continuation of the test compared to the reduction in cost of misclassification (both false positives and false negatives) that can be expected to result from administering additional items. Vos[28], Welch and Frick[29] and Rudner[30,31] have discussed the extension of decision theory to item-selection.

These testing methods, which *adapt *to the examinees' perceived ability in order to determine the item order and test length, can be suitably administered using computers. Computerized adaptive testing (CAT) provides a way to make assessment more comprehensive while limiting additional burden on the test-taker [26,32]. CAT is routinely used in standardized educational testing and has recently been applied to patient-oriented assessments in health and medicine. Mead and Drasgow's review of 123 tests showed that CAT and paper-and-pencil tests are equivalent for carefully constructed tests[33]. For instance, the Patient-Reported-Outcomes Measurement Information System (PROMIS) is an NIH Roadmap network project that uses CAT to improve the reliability, validity, and precision of patient-reported outcomes[34]. CAT has also been used successfully to screen for developmental problems [35], assess tobacco beliefs among young people [36] and to administer headache impact tests [37]. It has consistently been shown to be an efficient testing approach with high precision. We believe that in situations when the examinees need to be classified into relatively few categories, measurement decision theory (MDT) can provide comparable performance with significantly fewer items and a much smaller test population [30].

In this paper we discuss the application of a MDT-based approach to administer variable-length tests in order classify examinees into a limited number of discrete categories. Pilot testing with a relatively small calibration sample is used to estimate the conditional probabilities that subjects of particular literacy levels will answer a particular question correctly. Subsequently, Bayes' Theorem is used to compute the likelihood that an individual test-taker who answers a series of questions correctly is of a specified literacy level. We demonstrate the validity of the proposed method using data from two patient health literacy questionnaires.

## Methods

### Algorithm development

In the MDT-based CAT process, the goal is to place the examinee in one of *k *literacy classes (e.g., low or adequate; or low, adequate, or high). One item is presented at a time, and on the basis of the test-taker's previous answers, the next 'best' item is the item that eliminates the most uncertainty about the classification.

Specifically, as described below, the best question is the one that maximally reduces the entropy of the classification. Prior to testing, background or calibration data is needed to calculate:

1. *P(L_i_)*- the distribution of different competence levels (*L_i_*) in the participant population;

2. *P(Q_j _= 1/L_i_) *- the conditional probability that participants of a particular competence level *L_i _*respond correctly to a question *Q_j_*.

At each point in the testing process, the test-taker's current response vector is *Z *= *Z_0_Z_1_...Z_n_*, where *Z_j _*is 1 if the participant provided the correct response to *Q_j _*and 0 otherwise. According to Bayes' Theorem, the conditional probability that s/he belongs to competence level *L_i _*given the data vector *Z *is thus:(1)

where *'k' *is the number of possible classes. If questions are independent (a central assumption in MDT), then *P(Z/L_i_)*, the conditional probability of the response vector given competence level *L_i_*, is the product of the responses' individual conditional probabilities.(2)

The response vector also defines the current state of the participant, *S_curr_*. The information content of this state can be calculated as entropy formulated by Shannon's information theory [38]:(3)

After each answered question, the next question is selected such that the new state, *S_next _*resulting from the participant's response to it results in the greatest reduction in entropy i.e. the question that maximizes:(4)

In (4), *Z' *= *Z_0_Z_1_...Z_n_Z_n+1 _*can be one of two vectors depending on whether the participant answers *Q_n+1 _*correctly or incorrectly. If *Z'_1 _*is the vector resulting from a correct response and *Z'_0 _*the vector resulting from an incorrect response, for each of the unanswered questions, *Q_new _*the entropy of the state resulting from presenting the question is calculated as:(5)

Here *P (Q_new _= 1) *is the probability that *Q_new _*is answered correctly and is given by:(6)

*Termination: *The testing process could theoretically continue until the participant answered all available questions. However, to gain efficiency, we terminate testing if *H(S_next_) *(from 4) was less than a specified threshold for three successive questions. At termination, the participant's predicted class is the class *L_i _*with maximum *P(L_i_/Z)*.

### Materials

We validated our MDT-based CAT approach through a secondary analysis of data from two assessment tests developed for consumers in the health domain. The algorithm was applied retrospectively to the data sets to (a) determine an optimal question order and (b) to categorize each participant into a literacy or numeracy category. Finally, we also performed ROC analysis to characterize the sensitivity, specificity, and predictive power (area under the curve) of the algorithm.

#### Data Set 1

**Figure 1:**
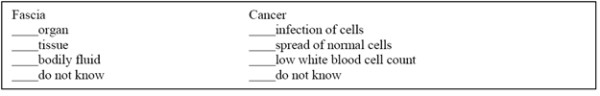
**Example of questions from study 1 that tested consumers' familiarity with terms ("fascia") and concepts ("cancer")**.

This paper-based questionnaire study [39] was originally conducted to assess patients' familiarity with health-related terms. The 52 participants were recruited from the Brigham and Women's Hospital, an academic medical center in Boston, MA (Table 1). Each participant was required to answer 60 questions that tested his/her familiarity with a term (n = 45) or concept (n = 15). The *target *terms for the questions were manually selected from consumer health terms identified in three frequently visited MedlinePlus articles related to hypertension, back pain and gastroesophageal reflux disease (GERD). For each question (Figure 1), the participant was invited to select one of the four available responses:

**Table 1:** Demographic characteristics of study samples

	Keselman	Ancker	
Characteristic	(n = 52)	Online (n = 100)	Clinic (n = 65)
Age bracket, n (%)			
18-25	5 (9.6)	33 (33.0)	26 (40.0)
26-39	13 (25.0)	40 (40.0)	26 (40.0)
40-59	25 (48.1)	26 (26.0)	11 (16.9)
≥ 60	9 (17.3)	1 (1.0)	2 (0.03)

Number (%) women	36 (69.2)	64 (64.0)	41 (63.1)

Educational level, n (%)			
no bachelor's degree	11 (21.0)	19 (19.0)	28 (45.0)
some college	20 (38.5)	37 (37.0)	23 (35.4)
bachelor's or graduate degree	21 (40.4)	44 (44.0)	14 (21.5)

Self-identity, n (%)			
African - American	13(25.0)	10 (10.0)	10 (15.4)
Asian	0	20 (20.0)	0
White	25 (48.1)	60 (60.0)	6 (9.2)
Hispanic	8 (15.4)	2 (2.0)	43 (66.2)
Other	6 (11.5)	3 (3.0)	3 (4.5)
Mixed race/ethnicity	0	5 (5.0)	3 (4.5)

Poor health literary (by S-TOFHLA), n (%)	0	1(1.0)	1(1.8)^a ^

^a^Missing S-TOFHLA scores for 8 clinic respondents because of interruptions during the test administration

1. a *key *term that was related to the *target *term in the question, either at the surface-level ("biopsy" is a "test") or at the concept level ("biopsy" means "removing sample of a tissue");

2. a *distractor *term that had the same semantic relation to the *target *term as the *key *term and of approximately the same difficulty as the *key *term;

3. a second *distractor *term satisfying the same criteria as 2;

*4*. a *do not know *option.

Results of the study are reported elsewhere [39].

Cronbach's alpha was found to be 0.93 signifying very high internal consistency. Factor analysis without rotation showed that all questions load heavily on one factor accounting for 26% of the variance. The second and third factors account for 10% and 7% respectively.

Different literacy measures use different cut-off points to assign participants to a category. For the current analysis, the participants' scores were used to classify them into one of three categories: *low literacy *(a score of 44 or lower), *moderate literacy *(a score in the range of 45 and 52) or *high literacy *(a score of 53 or higher). These score thresholds were selected for demonstration purposes so as to obtain approximately equal-sized groups: 34.6% of participants in the *low *literacy group and 32.7% in *moderate *and *high *literacy groups. However, different thresholds could be selected for other purposes.

#### Data Set 2

This on-line questionnaire study was conducted as part of a larger study to evaluate the effect of different visual illustrations of risk on patient decision-making[40]. Two samples of adult consumers (n = 165) were recruited: one on-line and one from clinics at New York-Presbyterian Hospital, an academic medical center in New York City (Table 1). As part of this study, participants were assessed for numeracy with a short scale modified from that of Lipkus et al [5]. The scale consists of 8 questions to assess comprehension of probabilities. Two of the questions are in multiple choice format and the rest are of fill-in-the-blank format(Table 2). In the Lipkus study [5], the first question in Table 2 was an unscored practice question; however, following the example of Schwartz et al [6] and Gurmankin et al [41,42], the same item was used here as a valid numeracy question. Internal reliability of this modified Lipkus scale was good (Cronbach's alpha = 0.70). A factor analysis using principal components extraction without rotation showed that all questions loaded heavily on one factor that accounted for 34.3% of the variance; the two multiple-choice questions loaded on a weak second factor for 17.0% of the variance, and the two most difficult fill-in-the-blank questions (numbers 1 and 8) loaded on a weak third factor that accounted for 13.3% of the variance. These results were extremely similar to those reported by Lipkus et al [5], who concluded that the scale measured a single construct of *numeracy *and that the weak second and third factors were probably artifacts of the measurement scale.

**Table 2:** Numeracy scale with percentages answering correctly in two studies

		Ancker		
		
Question	Lipkus et al (n = 463)	on-line (n = 100)	Clinic **(n = 62***^b^***)**	Total (n = 162)
1. Imagine that we flip a fair coin 1,000 times. What is your best guess about how many times the coin would come up heads?	*question not scored^a^*	74.0	66.1	71.0

2. Which of the following numbers represents the biggest risk of getting a disease? _ 1 in 100, _ 1 in 1000, _1 in 10	78.2	81.0	54.8	71.0

3. Which of the following numbers represents the biggest risk of getting a disease? _ 1%, _ 10%, _ 5%	83.8	92.0	80.6	87.7

4. If Person A's risk of getting a disease is 1% in ten years, and person B's risk is double that of A's, what is B's risk?	90.5	96.0	71.0	86.4

If Person A's chance of getting a disease is 1 in 100 in ten years, and person B's risk is double that of A's, what is B's risk?	86.6	*question not used*		

5. If the chance of getting a disease is 10%, how many people would be expected to get the disease out of 100?	80.8	95.0	67.7	84.6

6. If the chance of getting a disease is 10%, how many people would be expected to get the disease out of 1000?	77.5	89.0	61.3	78.4

7. If the chance of getting a disease is 20 out of 100, this would be the same as having a ____% chance of getting the disease.	70.4	94.0	53.4	78.4

8. The chance of getting a viral infection is .0005. Out of 10,000 people, about how many of them are expected to get infected?	48.6	60.0	32.3	49.4

^a^In a sample of 287 veterans who completed a mail questionnaire [6], 54% answered this question correctly.

^b^Does not include responses of three subjects who were interrupted while completing the online questionnaire; on restarting the test, an unexpected system malfunction prevented their responses from being captured.

For the current analysis, two categorization schemes were developed for demonstration purposes. In the first scheme, participants were categorized as *low numeracy *(a score of 5 or lower, n = 48 (29.1%)) or *high numeracy*; in the second scheme, they were categorized as *low numeracy *(a score of 5 or lower, n = 48(29.1%)), *moderate numeracy *(a score of 6 or 7, n = 74(44.8%)), or *high numeracy*. Classifying patients into 3 categories of competency is typical of other assessments such as the TOFHLA [3].

### Evaluation

The gold standard literacy or numeracy level, true category, of a participant was defined by his/her total score on the original questionnaire as described above. The MDT-CAT algorithm was applied to predict the true category of the participant using a leave-one-out approach. For example, for the first data set, data from 51 of the 52 participants served as calibration cases - i.e. used to estimate the distribution *P(L_i_) *and *P(Q_j _= 1|L_i_)* - and the 52nd participant served as the test case. The process was repeated so that each participant served as the test case exactly once.

To initialize the testing process, i.e. to determine the first question to be presented, the following two alternatives were explored:

***SA***: Question selection criteria (i.e. entropy minimization) described by (4), (5) and (6) by substituting *P(L_i_) *for *P(L_i_|Z) *(since *Z *is not initialized).

***SB***: Any question that a participant known to be of moderate literacy has a 40-60% chance of answering correctly.

The average number of questions that needed to be presented prior to termination and the number of wrong classifications were calculated and reported.

## Results

For data set 1, using the start criterion ***SA ***and a termination threshold of 0 (i.e. no termination threshold), the algorithm classified 88.5% (n = 46) of the participants correctly. Of the misclassified participants (n = 6), half were of moderate literacy incorrectly classified as low literacy while the others were of high literacy classified as moderate.

All the misclassified cases appear to result from the same type of outlier question/response pair. For instance, one of the misclassification resulted when a participant with a score of 45 was misclassified to be of low literacy (true category: moderate literacy). An analysis of the response vector and state entropies revealed that this can be attributed to the participant's response to one particular question, for which the participant gave an incorrect response, in contrast to all other participants of moderate literacy. As the algorithm uses a leave-one-out approach, P(Q_i _= 0|moderate literacy) for this question would be equal to 0, and the participant's incorrect response results in a misclassification.

The use of a random moderate-difficulty start question (start criterion ***SB***) did not result in any discernible difference in the number or type of errors produced by the algorithm.

**Figure 2:**
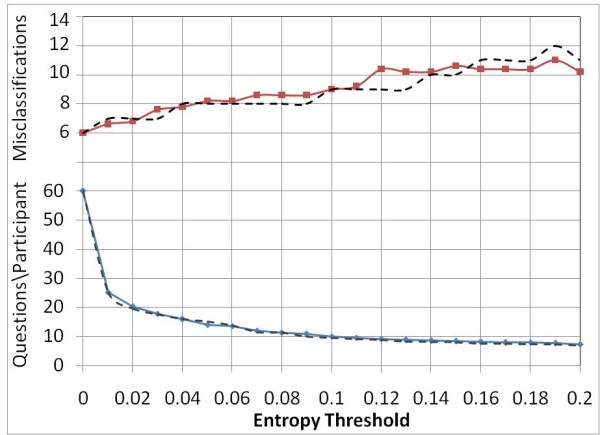
**Entropy threshold vs. Average question count and Error rate using start criterion SA (dotted lines) and SB(solid lines)**.

As expected, with an increase in the threshold the number of questions to be answered decreases and the error rate increases. Figure 2 shows the average number of question per participant and error rates for threshold values in the range of 0 and 0.2 in increments of 0.01. At the smallest attempted threshold of 0.01 the average number of questions to be answered was found to be 25 (42%) with one additional misclassification.

**Figure 3:**
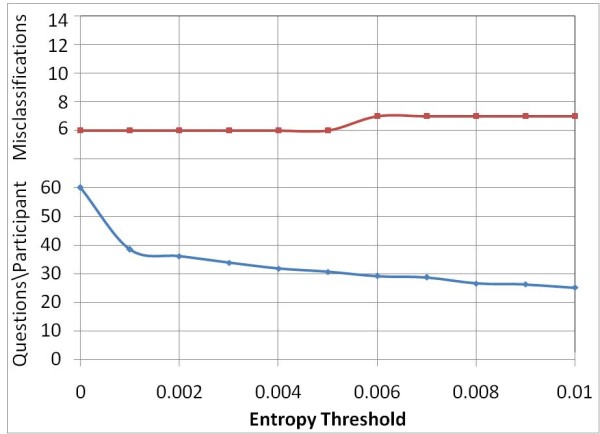
**Entropy threshold vs. Average question count and error count, for entropy thresholds in range 0 to 0.01 with start criterion *SA***.

To determine the largest possible reduction in questions without increasing errors, threshold values in the range of 0 and 0.01 were further investigated and the results are shown in Figure 3. A threshold value of 0.005 appears to be the most promising as this would reduce the number of questions by half without causing any additional misclassifications. For all participants, the predicted class at this threshold is identical to the predicted class when no termination criterion was specified.

Figure 2 also shows the result of using start criterion ***SB ***in combination with different entropy thresholds. As can be seen in the figure, the reduction in questions is comparable to our earlier results. However, the method tends to misclassify a slightly larger number of participants.

**Figure 4:**
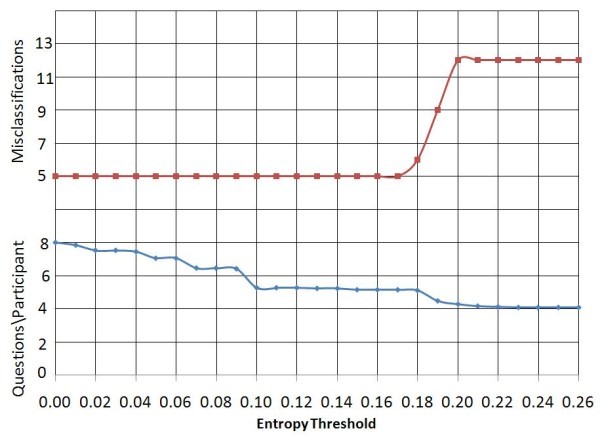
**Entropy threshold vs. Average question count and error count using start criterion SA, *for data set 2 with two-class classification scheme***.

Data set 2 has a larger number of subjects but a small item pool. Figure 4 shows the changes in the average number of questions to be answered and error rate for the two class classification scheme. Without a threshold, 96.9% of the participants were found to be correctly classified. The number of misclassifications remains constant with increase in threshold, up to an entropy threshold of 0.17 and the average number of questions to be answered reduces to 5.14 (64.25%).

**Figure 5:**
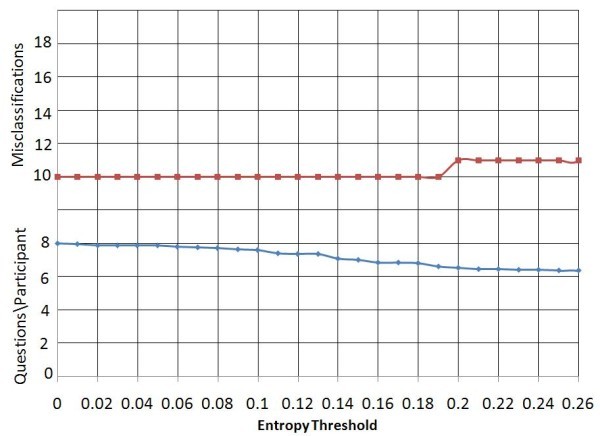
**Entropy threshold vs. Average question count and error count using start criterion *SA, for data set 2 with three-class classification scheme***.

A three-class classification can be expected to have more uncertainty than the two-class classification scheme, producing a higher error rate and a smaller reduction in question count. Figure 5 shows that for the three-class classification scheme on dataset 2, without a threshold, 93.8% of the subjects were correctly classified. Though this is less than the corresponding value in the two-class classification scheme, it is better than the observed errors of the three-class classification scheme on data set 1. The number of questions to be answered, 6.59 (82.3%), is also higher than that observed in the two-class classification scheme.

In order to estimate the sensitivity of the performance of the method to calibration data, we tried two calibration alternatives to the leave-one-out approach described above: (a) calibration with a random half of the population; (b) calibration using the online sample. In both cases, only subjects not used for calibration were used for testing. For example in scheme (b), *P(Q_j _= 1 | L_i_)* and *P(L_i_) *were computed using the subjects recruited online (n = 100) and used to classify subjects recruited at the clinic (n = 62). Table 3 and Table 4 list *P(Q_j _= 1 | L_i_)* and *P(L_i_) *observed in the three calibration schemes.

**Table 3:** Actual P(Q_j _= 1 | L_i_) for various subsets of Dataset 2 - complete sample, a random half of the sample, online sample (n = 100) and clinic sample (n = 62)

Q-ID	Class (L_i_)	Complete Sample	Random 50% split	Online Sample	Clinic Sample
	Low	0.5	0.43	0.56	0.47
1	Moderate	0.69	0.68	0.60	0.85
	High	1	1	1	1

	Low	0.31	0.35	0.25	0.34
2	Moderate	0.81	0.79	0.85	0.74
	High	1	1	1	1

	Low	0.69	0.70	0.63	0.72
3	Moderate	0.93	0.95	0.96	0.89
	High	1	1	1	1

	Low	0.60	0.61	0.81	0.5
4	Moderate	0.96	0.95	0.98	0.93
	High	1	1	1	1

	Low	0.56	0.61	0.87	0.41
5	Moderate	0.95	0.97	0.94	0.96
	High	1	1	1	1

	Low	0.5	0.48	0.69	0.41
6	Moderate	0.85	0.84	0.87	0.81
	High	1	1	1	1

	Low	0.4	0.43	0.75	0.22
7	Moderate	0.92	0.95	0.96	0.85
	High	1	1	1	1

	Low	0.13	0.17	0.06	0.16
8	Moderate	0.46	0.5	0.47	0.44
	High	1	1	1	1

**Table 4:** Actual P(L_i_) of the *calibration *sets for Dataset 2 using three different calibration schemes - leave-one-out, a random half of the sample, and online sample (n = 100)

Li	Complete sample	Random 50% split	Online sample	Clinic Sample
Low	0.29	0.28	0.16	0.52
Moderate	0.46	0.46	0.47	0.43
High	0.25	0.26	0.37	0.05

Using the leave one-out approach, the average number of questions to be answered was 6.6 at 93.8% classification accuracy (accuracy possible if no threshold were used). For calibration scheme (a), 6.8 questions needed to be answered and a higher accuracy of 97.5% was observed. Calibration scheme (b) resulted in a classification accuracy of 91.9% with the subject having had to answer 5.3 items on average. As can be seen in Table 4 for scheme (b), the calibration sample has a very different distribution from the testing sample. For example, in the online sample, 16% of the subjects were of low numeracy and 37% of high numeracy whereas in the clinic population 52% were of low numeracy and only 5% of high numeracy. The decrease in performance using scheme (b) can probably be attributed to this difference.

Table 5 shows the actual and predicted distribution of the numeracy classes in the testing sets of the three calibration schemes. The misclassifications resulted from overestimation of the numeracy of subjects of low numeracy group and when the calibration set is not representative of the testing sample, as in scheme (b), these misclassifications were found to increase.

**Table 5:** P(L_i_) predicted for the *testing *sets using the three calibration schemes

Li	Leave-one-out		Scheme (a)		Scheme (b)	
	Actual	Predicted	Actual	Predicted	Actual	Predicted
Low	0.29	0.23	0.31	0.29	0.52	0.43
Moderate	0.46	0.52	0.45	0.47	0.43	0.52
High	0.25	0.25	0.24	0.24	0.05	0.05

In scheme(a) the testing set only included subjects not used for calibration; in scheme (b) the testing set is the clinic sample.

**Figure 6:**
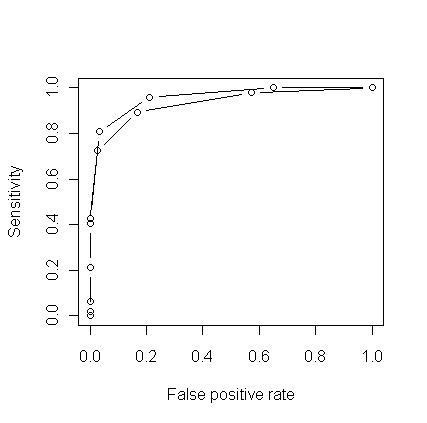
**Sensitivity and specificity of the algorithm for Data set 2 with two class classification scheme**. The top ROC curve shows the sensitivity and false positive rate on the numeracy data set at an entropy threshold of 0 (area under the curve = 0.96); the lower curve shows very little decrement in performance at an entropy of 0.6 (area under the curve = 0.93).

As illustrated in the ROC curve in Figure 6, the sensitivity and specificity of the algorithm are very good. The top ROC curve shows the sensitivity and false positive rate on the numeracy data set at an entropy threshold of 0 (area under the curve = 0.96); the lower ROC curve shows very little decrement in performance at an entropy of 0.6 (area under the curve = 0.93).

## Discussion

Comprehensive, robust and parsimonious diagnostic health literacy measures are needed in consumer health initiatives, as well as clinical practice, to identify low literacy individuals. The need is particularly acute in older adults who are more likely to require frequent health care services and are also more likely to experience fatigue and cognitive burden from a lengthy testing procedure.

Our results show that the method discussed in this paper can be useful in reducing the number of questions that need to be answered by a participant without seriously compromising classification accuracy. For the data described in this study, we found that by selecting an appropriate threshold, the number of questions can be reduced by half without making any additional misclassifications. In addition, this high degree of accuracy was achieved with only few calibration cases. By contrast, the number of cases needed for calibrating IRT-based algorithms is estimated to be quite large [25,30]. For demonstration purposes, we applied it to data sets from relatively brief assessments. Even with these short assessments, the algorithm markedly reduced the number of test items; the impact on our target population would be even more substantive if this algorithm were applied to lengthier competency assessments. Although MDT-based adaptive testing methodologies have been proposed and used in other domains, to the best of our knowledge this is its first application in the health domain.

The thresholds used to report the results are values at which the greatest reduction in test length was observed at the same classification accuracy as that achieved had no threshold been used. In a real testing scenario, these values can be estimated by simulating the leave-one out approach on the calibration sample. These simulations can also inform the test administrators on the accuracy/test-length trade-offs and help them choose higher thresholds that further reduce the length of the tests.

Results observed with calibration scheme (b) of the second dataset also suggest that the users of the method should be encouraged to have a calibration set that is representative of the overall population and if this is not possible the performance of the classification accuracy can be expected to decrease. Additional analysis, preferably with larger datasets, would be necessary to determine the response of the performance to deviations between calibration and testing samples.

Although the results are promising, this study has some limitations. First, it relies on secondary analysis of data from assessments that measure vocabulary familiarity and numeracy, constructs that are related to or contribute to health literacy but do not fully cover the domain of health literacy. Second, the MDT algorithm is predicated on the assumption that the responses to questions are independent of other responses, and this may not be valid. The effect of the violation of this assumption on the performance of this algorithm remains to be investigated. Future work could compare algorithm performance on assessments that have highly correlated questions and assessments with highly independent questions, with independence inferred from Cronbach's alpha or factor analysis. It is relevant to point out however, that IRT makes a similar assumption of question independence.

A final limitation is that the assessment would have to be administered on a computer, and many individuals with low literacy also have low computer literacy. We are currently developing a computer interface for this assessment that is expressly designed to be easier to use for our target population of older adults, many of whom are novice computer users. The processing/memory requirements to administer this kind of testing are minimal and a fully equipped computer is not necessary. Touch screen devices such as tablet computers - which are becoming more affordable - hold promise as a more user-friendly medium for administering these tests in healthcare settings.

In addition, in future work, we intend to study how to apply this method to scenario-based tests where each scenario is described and is followed by several related questions. In such cases, probability and entropy calculations may need to be performed at the module level rather than at the individual question level. Additionally, we intend to validate the method by applying it to directly test the subjects in contrast to the retrospective process used in this study.

## Conclusions

The computer-adaptive testing method presented in this paper, which is based on measurement decision theory, can significantly reduce the number of items needed to classify subjects into categories corresponding to levels of competency without significantly compromising the accuracy of the classification. In addition, the measure can be validated with few subjects and test items. This method creates the potential for the development of sensitive diagnostic instruments for measuring health literacy efficiently and without undue burden on subjects.

## Competing interests

The authors declare that they have no competing interests.

## Authors' contributions

QZT, DRK and SK designed and implemented the algorithm. All authors contributed to data analysis and manuscript preparation. All authors have read and approved the final manuscript.

## Pre-publication history

The pre-publication history for this paper can be accessed here:

http://www.biomedcentral.com/1472-6947/11/52/prepub
